# Modulation of Hepatic Glutamate Transporters by MC-100093 in Male and Female Alcohol-Preferring Rats Exposed to Ethanol

**DOI:** 10.3390/biomedicines14102250

**Published:** 2026-10-04

**Authors:** Ahmed Alotaibi, Khokon Kanti Bhowmik, Virender Kumar, Magid Abou-Gharbia, Wayne Childers, Youssef Sari

**Affiliations:** 1 Department of Pharmacology and Experimental Therapeutics, College of Pharmacy and Pharmaceutical Sciences, The University of Toledo, Toledo, OH 43614, USA; ahmed.alotaibi3@rockets.utoledo.edu (A.A.); khokonkanti.bhowmik@rockets.utoledo.edu (K.K.B.); 2 Department of Pharmacy Practice, College of Pharmacy and Pharmaceutical Sciences, The University of Toledo, Toledo, OH 43614, USA; virender.kumar@utoledo.edu; 3 Department of Pharmaceutical Sciences, Temple University School of Pharmacy, Philadelphia, PA 19140, USA; aboumag@temple.edu (M.A.-G.); wayne.childers@temple.edu (W.C.)

**Keywords:** MC-100093, alcohol-related liver disease, GLT-1, xCT, inflammation, hepatic-glutamate transporter

## Abstract

**Background/Objective:** Prolonged ethanol use can induce behavioral and physiological alterations and impair the liver’s capacity to metabolize ethanol, leading to hepatocyte injury. The spectrum of this disease ranges from fat accumulation to severe liver damage, including alcohol-related hepatitis and cirrhosis. MC-100093, a beta-lactam non-antibiotic compound, is known to modulate glutamate transporter-1 (GLT-1) and reduce ethanol drinking. Therefore, this study examined the effects of two optimal doses of MC-100093 on ethanol consumption and, consequently, ethanol-induced hepatic injury in both male and female alcohol-preferring (P) rats. **Methods:** Animals were divided into four groups and exposed to ethanol for six weeks. In week six, rats received intraperitoneal injections of saline, 100 mg/kg MC-100093, or 150 mg/kg MC-100093 for five consecutive days. Liver tissue samples were analyzed to assess GLT-1 and the cystine/glutamate antiporter (xCT), triglyceride levels, collagen type-1 (COL-1) deposition, and inflammatory cytokines. **Results:** MC-100093 effectively attenuated ethanol-induced liver damage by decreasing ethanol intake and restoring GLT-1, xCT, and COL-1 levels in the livers of both male and female P rats. Moreover, MC-100093 reversed ethanol-induced inflammation and reduced hepatic triglyceride levels. **Conclusions:** Our data show that MC-100093 effectively mitigates ethanol-induced liver injury.

## 1. Introduction

Chronic and excessive ethanol consumption may lead to significant neurological and hepatic complications [1,2,3]. It is well known that the liver metabolizes ethanol via several enzymes, including alcohol dehydrogenase and cytochrome P450 (CYP) 2E1 [4,5,6]. Besides this main oxidative pathway, ethanol is metabolized through non-oxidative processes involving conjugation reactions [7,8]. Chronic ethanol consumption can cause metabolic injury to the liver, which may progress from alcoholic fatty liver to inflammation and hepatocyte injury [9]. Alcohol liver injury involves the induction of oxidative stress, lipid peroxidation in hepatocytes, cytokine release by Kupffer cells (KCs), and activation of hepatic stellate cells (HSCs) to produce collagen type-I (COL-1) [10,11,12,13]. Glutamate, the primary excitatory neurotransmitter [14], is also involved in several metabolic pathways [15]. Importantly, glutamate is essential for the synthesis of glutathione, and acts as an antioxidant to reverse ethanol-induced liver injury [16]. Among the glutamate transporters responsible for maintaining extracellular glutamate concentrations is glutamate transporter 1 (GLT-1) [17], which is expressed in the liver [18]. Studies have shown that changes in glutamate transporter 1 (GLT-1) expression are associated with liver injury. Moreover, the expression of GLT-1 was increased in injured liver [19], in mice exposed to ethanol, and in patients with alcohol-related fatty liver (AFL) and alcohol-related steatohepatitis [20,21]. In addition, changes in the expression of cystine-glutamate antiporter (xCT) have been associated with oxidative stress in animal models involving ethanol liver toxicity. This oxidative stress may lead to liver steatosis and the development of ALD [5,14,22]. However, it remains unknown whether chronic ethanol exposure can alter hepatic GLT-1 or xCT, and whether such changes contribute to oxidative stress, steatosis, inflammation, or liver injury. Although hepatic GLT-1 has been implicated in other forms of liver injury, its role in alcohol-associated liver disease remains unclear [19,23]. Because xCT-mediated cystine uptake supports glutathione synthesis, modulation of GLT-1 and xCT could improve hepatic glutamate handling and antioxidant defenses during ethanol exposure [24,25,26].

A novel synthetic beta-lactam, MC-100093, which lacks antibiotic activity, has been shown to significantly modulate the imbalance in the expression of glutamate transporters in the brain [27,28]. In addition, MC-100093 can effectively attenuate ethanol consumption in alcohol-preferring (P) rats by modulating GLT-1 and glutamate uptake [28,29]. Furthermore, MC-100093 mitigated neuroinflammation resulting from chronic ethanol consumption in the brains of P rats [30,31] and decreased fat droplet content in the livers of male P rats [27]. This study explored the potential therapeutic effect of MC-100093 at two doses in the livers of female and male P rats exposed to ethanol. The effects of MC-100093 on the expression of GLT-1 and xCT in the liver were investigated. Furthermore, the expression of several inflammatory factors, tumor necrosis factor alpha (TNF-α), interleukin-6 (IL-6), interleukin-1β (IL-1β), and signal transducer and activator of transcription 3 (STAT3), as well as COL-1 deposition, were examined in the liver of female and male P rats. Histological studies were performed to assess the potential effects of MC-100093 on collagen deposition and inflammation in P rats exposed to ethanol for six weeks.

## 2. Materials and Methods

### 2.1. Animals

Ninety-day old male and female P rats were acquired from Indiana University School of Medicine, Indianapolis, IN. Each animal was housed individually in a room with free access to food and water throughout the experimental procedures. All the procedures in this experiment were in accordance with the guidelines of the University of Toledo IACUC and the National Institutes of Health (NIH).

### 2.2. Ethanol Drinking Paradigms and MC-100093 Treatment

Animals were randomly assigned to the following groups: a water-only control group and three ethanol-exposed groups. Rats in ethanol groups received continuous and had access to free-choice ethanol (15% and 30%, *v*/*v*, concurrently) and water bottles for 6 weeks. Consumption of ethanol was measured in grams per kilogram of body weight per day (g/kg/day). To ensure consistent exposure intensity, standardized intake calculations were used, and bottle positions were rotated to avoid side preference. Rats that failed to achieve an average intake of 4 g/kg/day were eliminated from the study [32,33]. At week six of the experiment, rats in the water-only group and one ethanol group received intraperitoneal (i.p.) saline injections (control water group and EtOH group), whereas the remaining two groups received either 100 or 150 mg/kg i.p. injection of MC-100093 [EtOH/MC-100093 (100 mg/kg); EtOH/MC-100093 (150 mg/kg), respectively] every consecutive day for five days. These two different doses were selected to examine the dose-dependent pharmacological effects of MC-100093 and to determine whether greater systemic exposure would produce stronger modulation of ethanol drinking and associated molecular outcomes. The five-day dosing regimen in week six was selected based on a well-established protocol for administering beta-lactams, such as ceftriaxone, to alcohol-preferring (P) rats using the same ethanol paradigm. Moreover, the five-day treatment is the known period for beta-lactam compounds to modulate GLT-1 expression and downstream signaling pathways [27,29,32,34].

### 2.3. Histological Analyses

Rats were euthanized by CO_2_ inhalation 24 h after the last i.p. injection of MC-100093. Liver tissues from different animal groups were fixed in 10% formaldehyde for 24 h and then placed in 70% ethanol before embedding in paraffin. Sections of liver tissues (5 μm thick) were stained with Hematoxylin and Eosin (H&E) and Masson’s trichrome (MT) following standard protocols. Sections were scanned using Olympus VS120 Slide Scanner Slide Analyzer at 40×. For quantitative analysis of collagen deposition, four microscopic fields for each specimen were quantified using ImageJ (Version 1.53t 24) to measure the relative collagen-positive area [35].

### 2.4. Western Blotting

Western blots were used to determine the expression of GLT-1, xCT, COL-1, and GAPDH as previously described [28]. Briefly, a lysing buffer supplemented with phosphatase and protease inhibitors was used for the homogenization of liver tissues, and total protein in all samples was quantified using BSA (Bio-Rad, Hercules, CA, USA). For each sample, an equal amount of protein was loaded onto an acrylamide gel. Next, proteins were transferred onto a polyvinylidene difluoride (PVDF) membrane and subsequently blocked for 30 min using 5% milk in Tris-buffered saline supplemented with Tween-20 (TBST) at ambient temperature. Afterward, the membranes were incubated at 4 °C for one night with the following antibodies: rabbit anti-GLT-1 (1:1000, Ab41621, Abcam, Waltham, MA, USA), rabbit anti-xCT (1:1000, Ab175186, Abcam), anti-COL-I (1:1000, Ab260043, Abcam), and the loading control antibody anti-GAPDH (1:1000, D16H11, Cell Signaling Technology, Danvers, MA, USA). Membranes were subsequently rinsed five times with TBST the following day, then incubated with a secondary antibody (1:4000) for 1 h at room temperature. Band densities were detected using chemiluminescent reagents [Super Signal West Pico (Pierce Inc., Appleton, WI, USA)] and the ChemiDoc imaging system (Bio-Rad, Hercules, CA, USA). GAPDH was used to normalize protein expression levels (GLT-1, xCT, and COL-1). Water group data were normalized to 100%, with all other values expressed relative to this control to evaluate changes in protein expression.

### 2.5. ELISA Assay and Measurement of Hepatic Triglyceride Concentrations

Hepatic inflammatory factors (TNF-α, IL-1β, IL-6, and phospho-STAT-3) were measured with ELISA kits (R&D system, Minneapolis, MN, USA). Hepatic triglyceride (TG) concentrations in liver tissues were determined by a TG assay kit following manufacturer instructions (Cayman, Ann Arbor, MI, USA).

### 2.6. Statistical Analysis

GraphPad Prism 10 was used to analyze all data. A two-way ANOVA with Tukey’s post hoc test was used to analyze mean daily ethanol consumption, water intake, and body weight changes. The analysis included the main effects of treatment, day, and treatment x day interaction. Western blot band densities in treated livers were analyzed with One-way ANOVA and Tukey’s test. Inflammatory cytokines and triglycerides were analyzed with one-way ANOVA and Tukey’s test. Significance was set at *p* < 0.05.

## 3. Results

### 3.1. Treatment Effects of MC-100093 on Ethanol Consumption, Water Intake, and Body Weight in Male and Female P Rats

#### 3.1.1. Ethanol Drinking

Average ethanol intake was measured in g/kg body weight per day after 5 consecutive days of i.p. injections of saline, MC-100093 (100 mg/kg), or MC-100093 (150 mg/kg). To determine the sex effect on ethanol drinking, a three-way ANOVA was performed. At both 100 mg/kg and 150 mg/kg (i.p.) doses, there was no significant main effect of Sex [100 mg/kg: F_(1, 16)_ = 4.361, *p* = 0.0531; 150 mg/kg: F_(1, 16)_ = 0.2438, *p* = 0.6282]. No significant Sex × Treatment interactions or Sex × Day interactions were observed at either dose.

**Figure 1:**
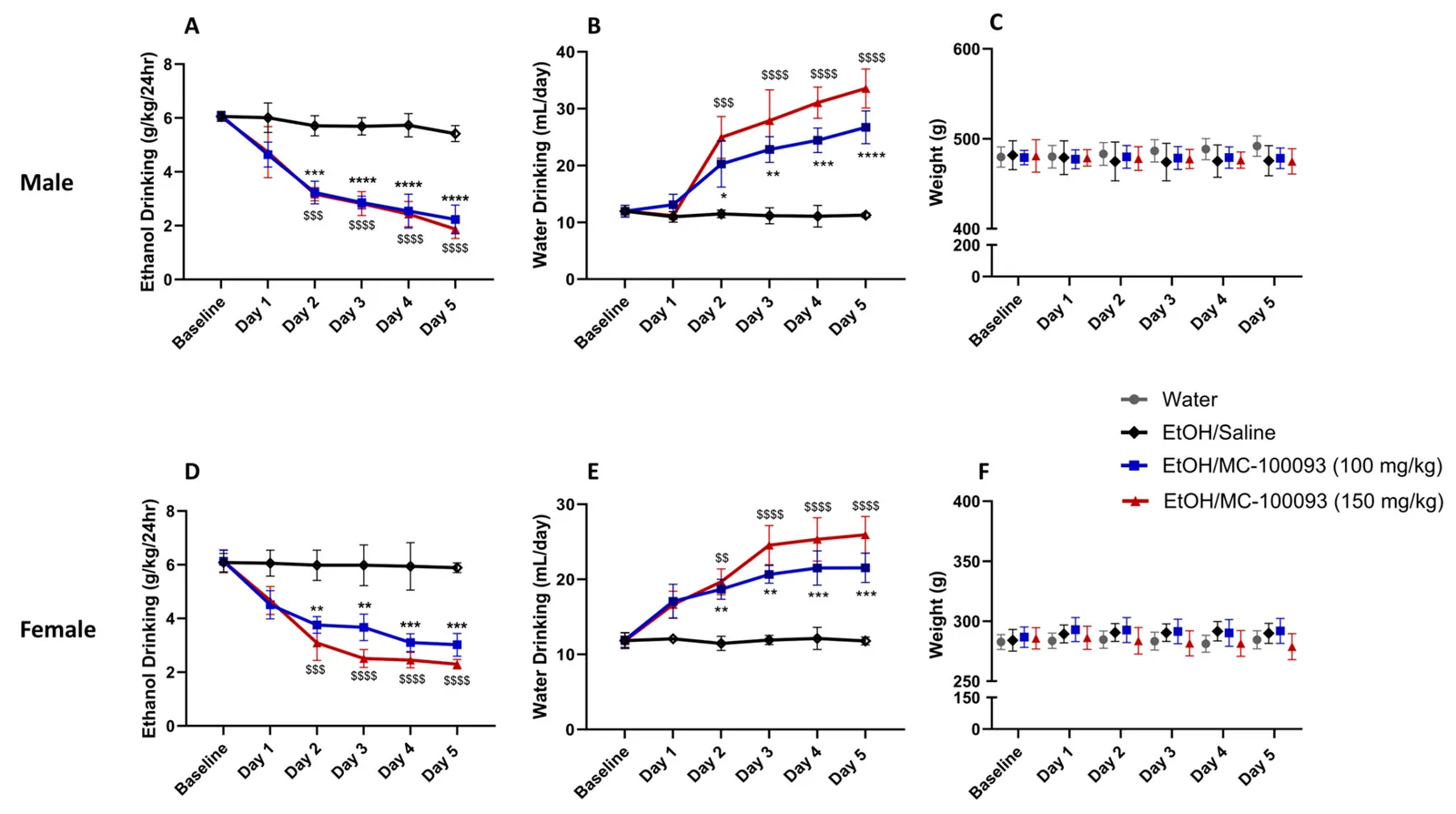
MC-100093 treatment effects on ethanol intake, water intake, and body weight in both sexes of P rats. (**A**,**D**) daily average ethanol intake (g/kg/day), (**B**,**E**) water consumption (mL/day), and (**C**,**F**) body weight (g) of male and female P rats treated for 5 days with 100 mg/kg or 150 mg/kg MC-100093, or saline. Statistical analysis showed that the MC-100093-treated group had significantly lower ethanol intake than the ethanol-saline-treated group. This effect of MC-100093 was significant from day 2 through day 5 compared with ethanol-treated rats receiving saline. Similarly, MC-100093 was associated with a significant increase in water drinking from day two through day five. No change in rat body weight was observed across all treatment groups. [mean ± SEM (n = 5/group)]. (* compares the difference between the ethanol saline group and the ethanol MC-100093 100 mg/kg drinking; $ represents the difference between the ethanol saline group and the ethanol MC-100093 150 mg/kg) (* *p* < 0.05, $$ ** *p* < 0.01, $$$ *** *p* < 0.001, and $$$$ **** *p* < 0.0001).

Next, a separate Two-way ANOVA within each sex was conducted to further characterize the treatment effects. Treatment with MC-100093 decreased ethanol intake in male P rats (Figure 1A). Two-way ANOVA showed significant effects of Treatment [F_(2, 72)_ = 50.57, *p* < 0.0001] and Day [F_(5, 72)_ = 18.30, *p* < 0.0001]. A significant Treatment × Day interaction was also observed [F_(10, 72)_ = 2.979, *p* = 0.0034]. Analysis with Tukey’s showed a significant reduction in ethanol intake in rats treated with MC-100093 at doses of 100 mg/kg and 150 mg/kg on day 2 (*p* = 0.0005; *p* = 0.0003, respectively), with further reductions on days 3, 4, and 5 (*p* < 0.0001).

Similarly, in female P rats (Figure 1D), analysis showed a significant Treatment × Day interaction [F_(10, 72)_ = 2.587, *p* = 0.0097] and significant effects of treatment and day on ethanol drinking [F_(2, 72)_ = 43.28, *p* < 0.0001; F_(5, 72)_ = 10.79, *p* < 0.0001]. A statistically significant reduction in ethanol drinking was observed in rats treated with MC-100093 at a dose of 100 mg on days 2 and 3 (*p* < 0.01) and on days 4 and 5 (*p* < 0.001) compared to the ethanol group. MC-100093 (150 mg/kg) reduced ethanol intake on day 2 (*p* = 0.0051) and from days 3 through 5 (*p* < 0.0001).

#### 3.1.2. Water Intake and Body Weight

The average water intake was calculated as (mL/day). The treatment with MC-100093 significantly increased water intake in male (Figure 1B) and female P rats (Figure 1E) compared to the saline group. Analysis using Two-way ANOVA showed a significant Day × Treatment interaction [F_(10, 72)_ = 4.033, *p* = 0.0002], a significant Treatment effect [F_(2, 72)_ = 38.10, *p* > 0.0001], and a significant Day effect [F_(5, 72)_ = 13.21, *p* > 0.0001] on water consumption in male P rats. Furthermore, female P rats showed a significant Day × Treatment interaction effect [F_(10, 72)_ = 2.931, *p* = 0.0041], as well as significant main effects of Treatment and Day on water intake [F_(5, 72)_ = 10.15, *p* < 0.0001; F_(2, 72)_ = 44.24, *p* < 0.0001]. Analysis with Tukey’s test showed a significant rise in water drinking among male P rats given MC-100093 at 100 mg/kg (*p* > 0.05) and 150 mg/kg (*p* > 0.001) on day 2. Similarly, female P rats also showed a significant increase in water intake in both MC-100093 groups (*p* > 0.01). Further increases in water drinking were seen in these groups on days 3, 4, and 5 compared to the ethanol-saline group. Moreover, treatment with MC-100093 did not have any effect on rat’s body weight (g) across all treatment days in male (Figure 1C) and female (Figure 1F).

### 3.2. Treatment Effects of MC-100093 on Hepatic Glutamate Transporters Expression in Male and Female P Rats Exposed to Ethanol

**Figure 2:**
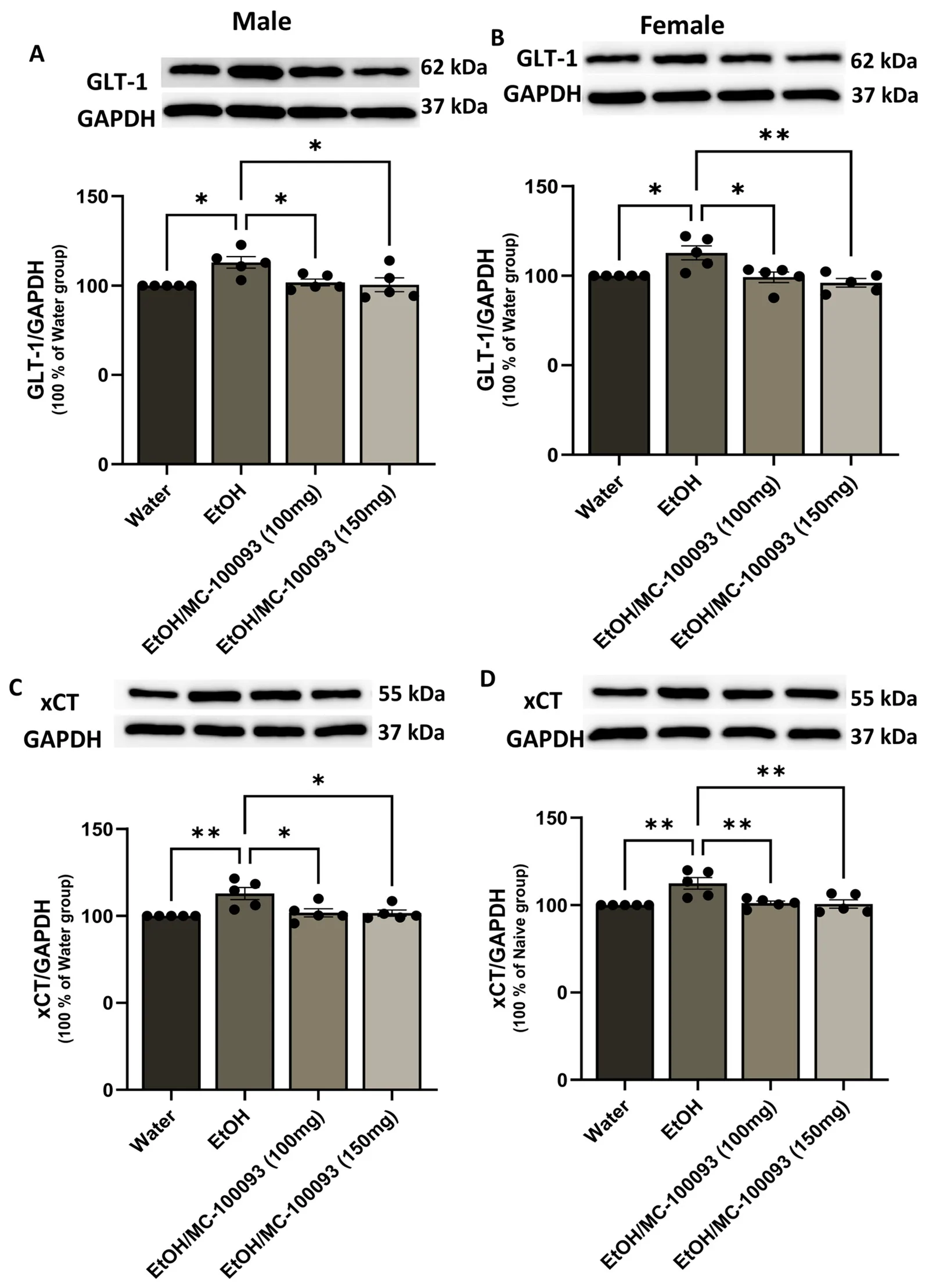
Treatment with MC-100093 changes the liver expression of GLT-1 and xCT. GLT-1 protein expressions were determined in (**A**) male and (**B**) female P rats after treatment with MC-100093. One-way ANOVA analysis showed that ethanol consumption increased the expression of GLT-1 (*p* < 0.05) in the liver of male and female P rats. (**C**,**D**) Similarly, xCT protein expression was upregulated in both male and female P rats’ livers (*p* < 0.01). Treatment with MC-100093 at 100 mg/kg and 150 mg/kg reversed ethanol’s effects and normalized expression of GLT-1 and xCT in the liver. The water group data is shown as 100%. Data expressed as mean ± SEM, (* *p* < 0.05, ** *p* < 0.01, *** *p* < 0.001, and **** *p* < 0.0001), (n = 5/group).

Hepatic GLT-1 and xCT expression in male and female P rats after six weeks of ethanol, with or without MC-100093, was measured by Western blotting. Protein expression analysis revealed that GLT-1 expression increased in the livers of male and female P rats exposed to ethanol (Figure 2A,B). Similarly, xCT expression was increased in the liver of both sexes following ethanol consumption (Figure 2C,D). Importantly, both doses of MC-100093 reversed the effect of ethanol and decreased GLT-1 and xCT in the liver of P rats of both sexes.

Statistical analysis displayed a significant treatment effect on GLT-1 expression in the livers of male rats across all groups (water, ethanol-saline, and ethanol-MC-100093) [F_(3, 16)_ = 5.246, *p* = 0.0103] (Figure 2A), as well as in female rats [F_(3, 16)_ = 7.314, *p* = 0.0026] (Figure 2B). Post hoc analysis using the Newman–Keuls test demonstrated a significant increase in GLT-1 expression in the ethanol-saline group compared to the water group (*p* < 0.05) in both male and female P rats. MC-100093 significantly decreased GLT-1 expression (*p* < 0.05) in male rats, with normalization observed at both doses. Similarly, MC-100093 decreased ethanol-induced upregulation of GLT-1 in the livers of female P rats at doses of 100 mg/kg (*p* < 0.05) and 150 mg/kg (*p* < 0.01). Statistical analyses revealed that ethanol consumption increased xCT expression in the liver of male and female of P rats (*p* < 0.01), whereas treatment with MC-100093 normalized the expression of xCT in the liver of both male (*p* < 0.05) (Figure 2C) and female (*p* < 0.01) (Figure 2D) P rats, and a significant treatment effect [F_(3, 16)_ = 6.721, *p* = 0.0038; F_(3, 16)_ = 7.835, *p* = 0.0019, respectively]. Both doses of MC-100093 showed no difference in their effects on xCT and GLT-1 in male and female P rats.

### 3.3. Treatment Effects of MC-100093 on Hepatic Triglyceride (TG) in Male and Female P Rats Exposed to Ethanol

**Figure 3:**
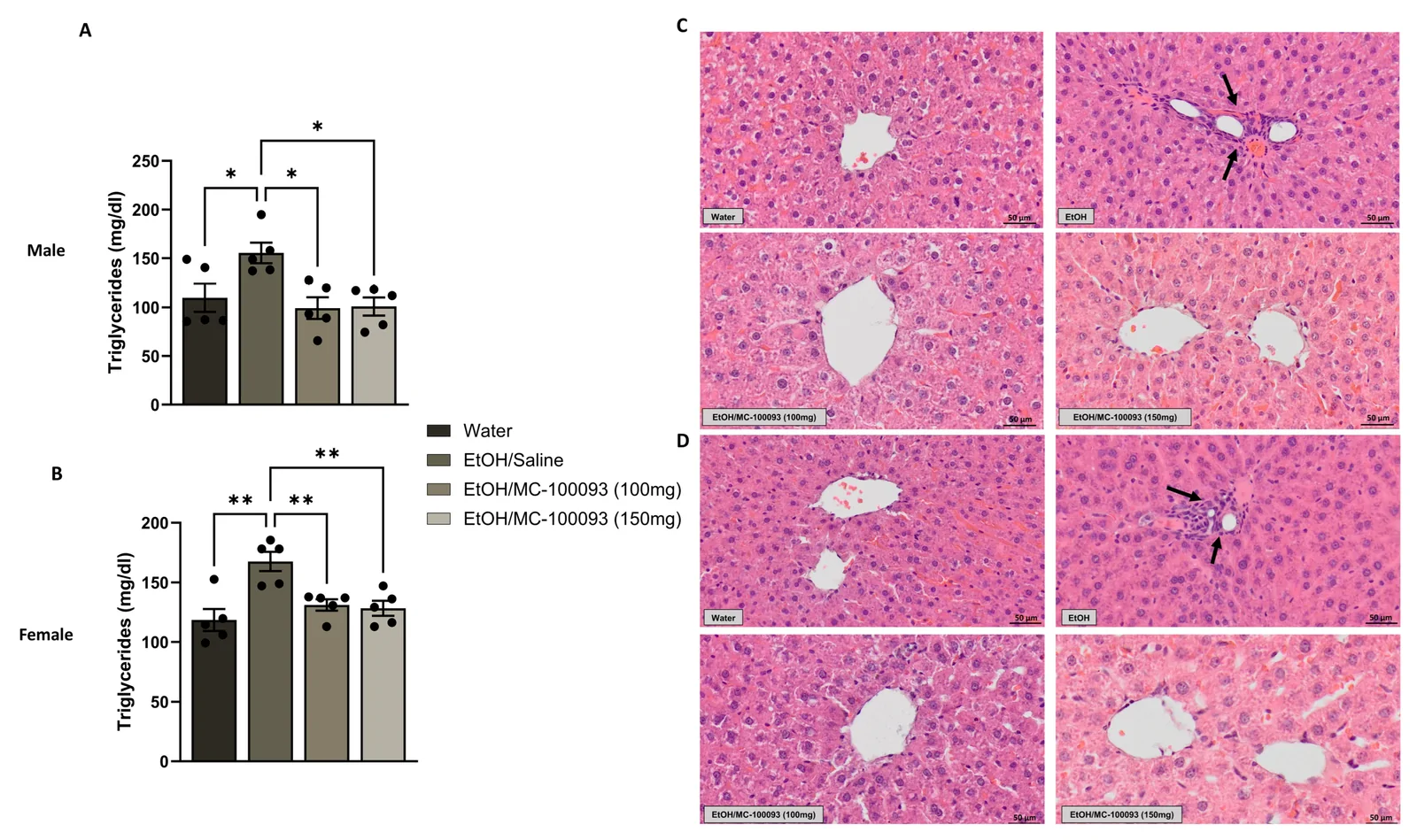
Treatment effects of MC-100093 on triglyceride (TG) concentrations in the liver of male and female P rats. Six weeks of ethanol consumption increased the TG concentrations in the liver of (**A**) male (*p* < 0.05) and (**B**) female (*p* < 0.01) P rats. Both doses of MC-100093 decreased the ethanol-induced increase in the TG concentrations in male (*p* < 0.05) and female (*p* < 0.01) livers. Representative images of H&E staining in (**C**) male and (**D**) female liver sections of water saline-treated group, ethanol saline-treated group [EtOH], ethanol MC-100093 100 mg/kg treated group [EtOH/MC-93 (100 mg)], and ethanol MC-100093 150 mg/kg treated group [EtOH/MC-93 (150 mg)]. Black arrows indicate foci of inflammation. (Magnification 20×, Scalebar 50 µm). Data expressed as mean ± SEM, (* *p* < 0.05, ** *p* < 0.01), (n = 5/group).

In a previous study from our laboratory, liver Oil Red O (OR) staining showed that ethanol consumption induced lipid droplet accumulation, which was attenuated by MC-100093 at 50 mg/kg [27]. Here, the study further investigated whether ethanol drinking and MC-100093 treatment at 100 mg/kg and 150 mg/kg affect triglyceride (TG) levels in the liver tissues of male and female P rats. Exposure to ethanol led to a significant increase in the liver TG levels in both sexes [F_(3, 16)_ = 5.312, *p* = 0.0099; F_(3, 12)_ = 8.680, *p* < 0.0044, respectively] (Figure 3A,B). MC-100093 significantly decreased the ethanol-induced increase in TG concentrations in both male and female P rats (*p* < 0.05 and *p* = 0.0012, respectively).

### 3.4. Treatment Effects of MC-100093 on Liver Injury and Inflammatory Markers in Male and Female P Rats Exposed to Ethanol

To investigate the effect of MC-100093 in reversing ethanol-induced liver injury and inflammation, liver sections were stained with H&E. Six weeks of ethanol exposure resulted in liver injury and infiltration of neutrophils, which were reversed by treatment with MC-100093, as shown by improved hepatic architecture in male (Figure 3C) and female P rats (Figure 3D).

**Figure 4:**
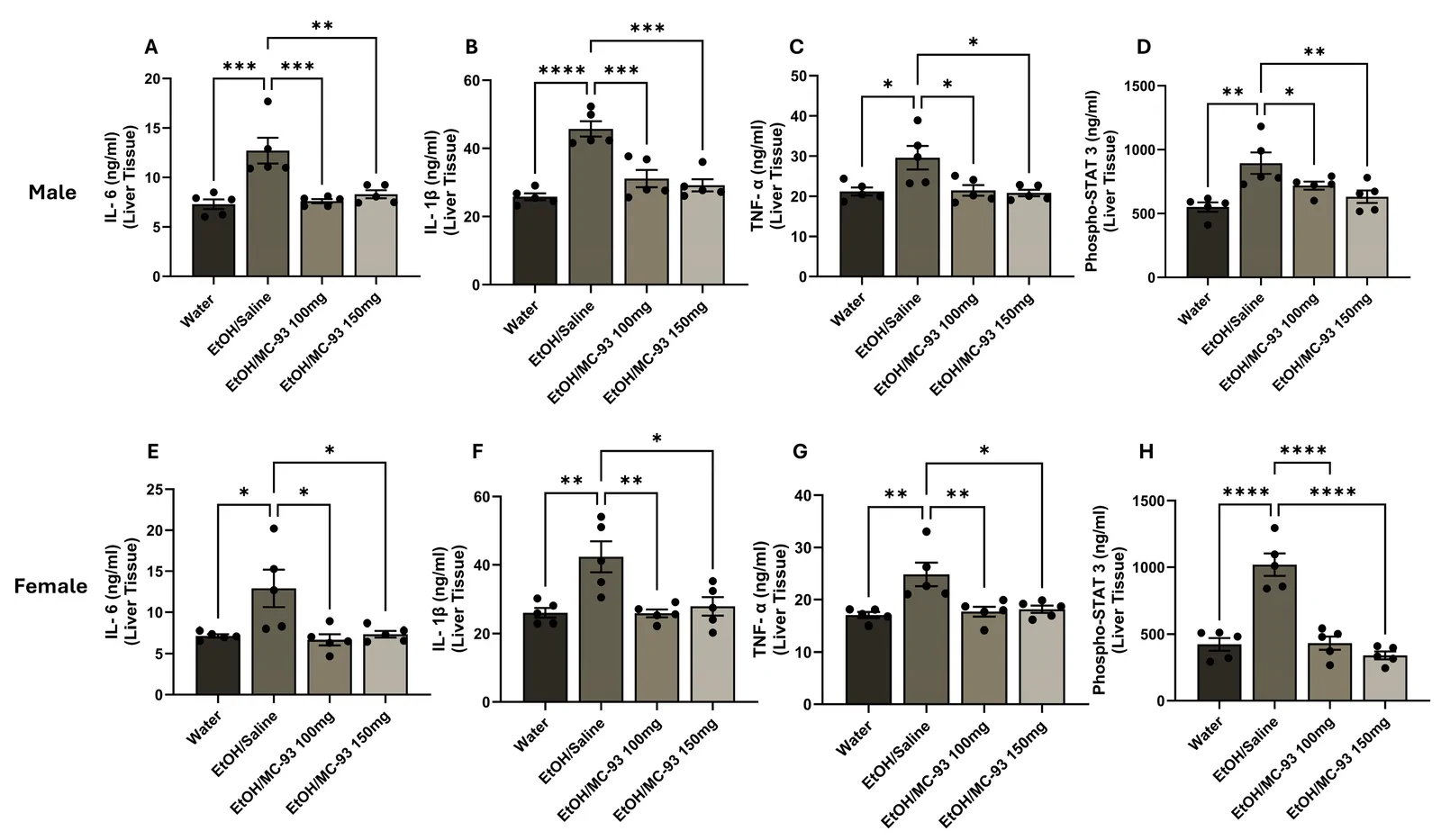
Treatment effects of MC-100093 on inflammatory cytokines in the liver of male and female P rats. Concentrations of IL-6 (**A**,**E**), IL-1β (**B**,**F**), TNF-α (**C**,**G**), and P-STAT3 (**D**,**H**) were determined using ELISA assays. Six weeks of ethanol consumption increased the concentrations of IL-6, IL-1β, P-STAT3, and TNF-α in the liver of male and female P rats. Treatment with MC-100093 significantly decreased the concentrations of inflammatory cytokines in the liver. Data expressed as mean ± SEM, (* *p* < 0.05, ** *p* < 0.01, *** *p* < 0.001, and **** *p* < 0.0001), (n = 5/group).

Given these findings, the effects of six weeks of ethanol exposure and MC-100093 treatment on the liver inflammatory markers were examined. ELISA assays showed that ethanol exposure resulted in a significant increase in hepatic inflammatory cytokines such as IL-6 (*p* < 0.001) (Figure 4A), IL-1β (*p* < 0.0001) (Figure 4B), TNF-α (*p* < 0.05) (Figure 4C), and p-STAT-3 (*p* < 0.01) (Figure 4D) compared to the control water group in male P rats. Similarly, in female P rats, six weeks of ethanol exposure increases the inflammatory markers as such IL-6 (*p* < 0.05) (Figure 4E), IL-1β (*p* < 0.01) (Figure 4F), TNF-α (*p* < 0.01) (Figure 4G), and p-STAT-3 (*p* < 0.0001) (Figure 4H) as compared to control water group. Whereas treatment with MC-100093 at both doses significantly reduced the concentrations of these inflammatory markers in the liver of P rats of both sexes.

### 3.5. Treatment Effects of MC-100093 on Collagen Deposition in Male and Female P Rats Exposed to Ethanol

**Figure 5:**
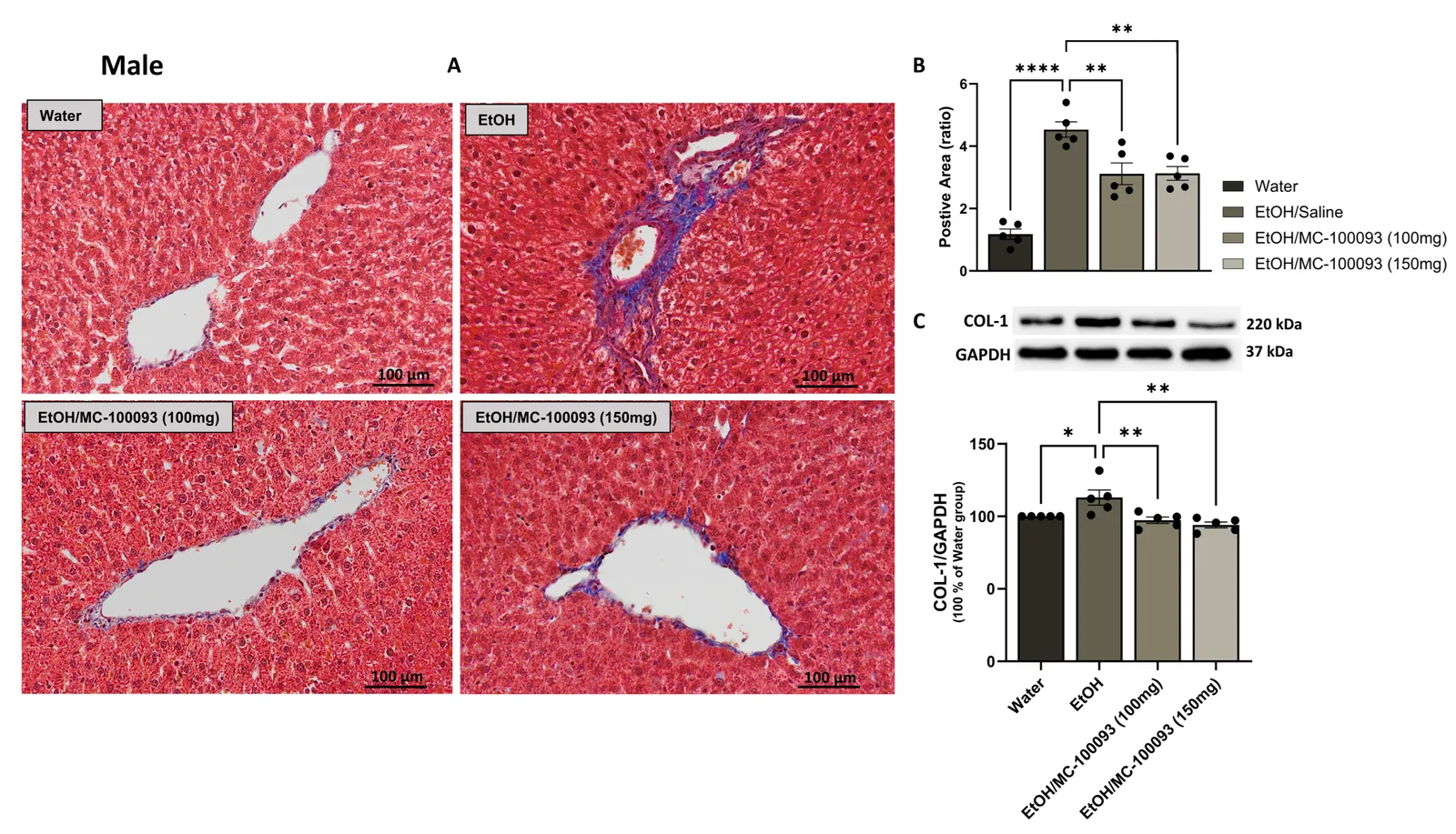
Treatment effects of MC-100093 on collagen deposition and COL-1 expression in the liver of male P rats. (**A**) Representative images of male liver sections stained with Masson’s trichrome (MT) for detecting collagen deposition. Section view showing collagen positive area of water saline-treated group, ethanol saline-treated group [EtOH], ethanol MC-100093 100 mg/kg treated group [EtOH/MC-100093 (100 mg)], and ethanol MC-100093 150 mg/kg treated group [EtOH/MC-100093 (150 mg)]. (**B**) Quantification of Masson’s trichrome staining using ImageJ software. Quantitative analysis revealed that six weeks of ethanol consumption increased the deposition of collagen (*p* < 0.05) in the liver of male P rats, and MC-100093 treatment decreased collagen positive area. (**C**) Treatment with MC-100093 reversed the effect of ethanol on the expression of COL-1 proteins in the liver, as determined by Western blot. Data expressed as mean ± SEM, (* *p* < 0.05, ** *p* < 0.01, *** *p* < 0.001, and **** *p* < 0.0001), (n = 5/group), (Magnification 20×, Scalebar 100 µm).

**Figure 6:**
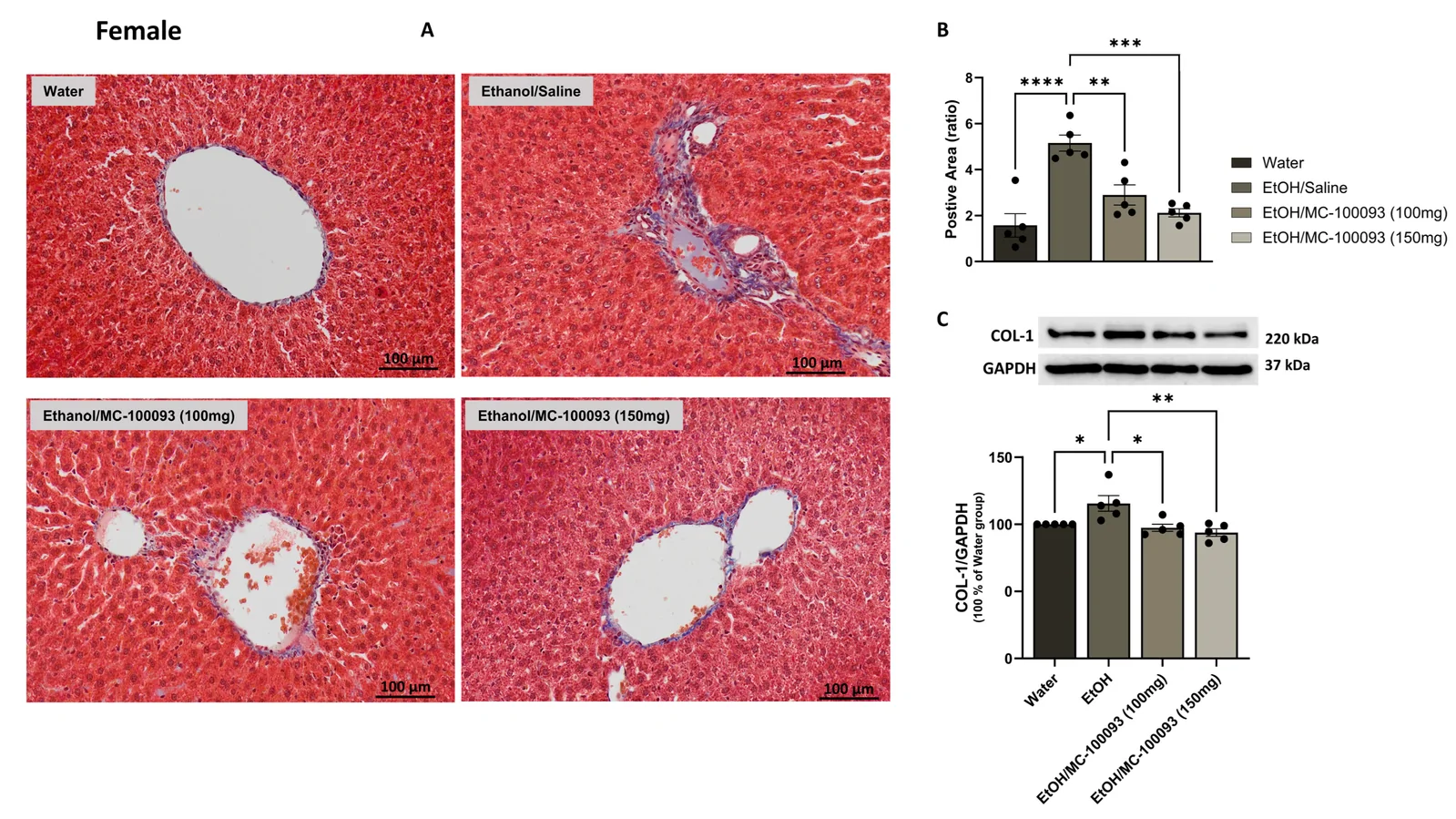
Treatment effects of MC-100093 on collagen deposition and COL-1 expression in the liver of female P rats. (**A**) Representative images of female liver sections stained with Masson’s trichrome (MT) for detecting collagen deposition. Section view showing collagen positive area of water saline-treated group, ethanol saline-treated group [EtOH], ethanol MC-100093 100 mg/kg treated group [EtOH/MC-100093 (100 mg)], and ethanol MC-100093 150 mg/kg treated group [EtOH/MC-100093 (150 mg)]. (**B**) Quantification of Masson’s trichrome staining using ImageJ software. Quantitative analysis revealed that six weeks of ethanol consumption increased the deposition of collagen (*p* < 0.05) in the liver of female P rats, and MC-100093 treatment decreased collagen positive area. (**C**) Treatment with MC-100093 reversed the effect of ethanol on the expression of COL-1 proteins in the liver, as determined by Western blot. Data expressed as mean ± SEM, (* *p* < 0.05, ** *p* < 0.01, *** *p* < 0.001, and **** *p* < 0.0001), (n = 5/group), (Magnification 20×, Scalebar 100 µm).

Chronic alcohol exposure activates hepatic stellate cells (HSCs), which initiate the production of extracellular matrix proteins, specifically collagen [36]. Therefore, collagen deposition was evaluated by Masson’s trichrome (MT) staining and COL-I protein levels to assess the effects of MC-100093 on liver fibrosis following 6 weeks of ethanol exposure. Ethanol exposure significantly increased the collagen deposition near the central vein in the liver sections of both male and female rats. Treatment with MC-100093 significantly reduced the collagen deposition area following a dose of 100 and 150 mg/kg of MC-100093 in male (*p* < 0.01) (Figure 5A,B) and female (*p* < 0.01, *p* < 0.001, respectively) (Figure 6A,B) P rats. Statistical analyses of COL-I expression revealed a significant effect of MC-100093 treatment in both sexes [male: F_(3, 16)_ = 7.590, *p* = 0.0022, (Figure 5C); and female: F_(3, 16)_ = 7.476, *p* = 0.0024, (Figure 6C)]. Ethanol markedly increased COL-I expression compared with the control water group (*p* < 0.05) in both sexes. Importantly, both doses of MC-100093 treatment reversed the ethanol-induced increase in COL-I expression in the liver of males (*p* < 0.01) and in females [(*p* < 0.05) with 100 mg/kg and (*p* < 0.01) with 150 mg/kg].

## 4. Discussion

Alcohol Use Disorder (AUD) has many adverse effects on various organs in the body, particularly the liver [1]. ALD is associated with steatosis, which can lead to hepatitis and cirrhosis [37,38]. In this study, six weeks of ethanol consumption resulted in liver injury and increased inflammatory response. Importantly, MC-100093 treatment reduced ethanol consumption and decreased the inflammatory response associated with the liver injury, which ultimately reversed ethanol-induced liver injury. Moreover, the data from this study suggest no sex differences in P rats and that MC-100093 had a similar effect on both sexes.

CYP2E1 metabolizes ethanol, thereby generating oxidative stress that triggers inflammation and hepatic cell damage [39,40,41]. Alcohol-induced liver damage is connected to excessive production of reactive oxygen species (ROS) associated with oxidative stress mainly within hepatocytes [27,42]. Glutathione regulates ethanol-induced oxidative stress in the liver [22,43]. In a chronic ethanol regimen, hepatic glutathione is depleted as ethanol consumption continues to generate ROS [10,44]. The synthesis of glutathione requires two main precursors, glutamate and cysteine, and depends on the methionine cycle as a source for cysteine [45]. The resulting cysteine shortage has been associated with increased xCT expression as a compensatory response that facilitates the exchange of extracellular cystine for intracellular glutamate [13,22]. Cystine is then reduced to cysteine, the rate-limiting precursor for glutathione synthesis, thereby linking xCT function to antioxidant function [25,46]. Moreover, studies have shown that ethanol exposure can promote oxidative stress, and this effect may be associated with depletion of hepatic glutathione and an impaired redox balance [47,48]. Additionally, an excess of extracellular glutamate may stimulate metabotropic glutamate receptor 5 (mGluR5) on HSCs, leading to subsequent activation of signaling cascades that result in steatosis [13,22] and liver fibrosis [49].

In this study, six weeks of ethanol consumption increased xCT expression in the liver, an effect also associated with increased GLT-1. The observed increase in GLT-1 expression might be considered as an adaptive mechanism to excess of glutamate, lipid accumulation, and oxidative stress mediated by increased xCT. GLT-1 is a key protein responsible for the uptake of glutamate, an important neurotransmitter involved in various central nervous system functions [17]. The increase in expression of GLT-1 in the liver is closely related to liver injury. A study reported increased expression of GLT-1 in the AFL and ASH patients at both the gene and protein levels [21]. Similarly, GLT-1 expression increased in mice exposed to ethanol [21]. The increase in GLT-1 expression following liver injury has been suggested to reflect a compensatory mechanism or a response associated with oxidative stress [19]. Moreover, elevated GLT-1 protein expression has also been observed in conditions characterized by cholestasis [23], providing further insights into the role of GLT-1 in the liver. This link between GLT-1 expression and liver injury highlights its potential role in the development and progression of liver diseases. In this investigation, MC-100093 reduced elevated levels of GLT-1 and xCT expression in the liver induced by ethanol. Thus, normalizing their protein expression can restore glutamate homeostasis in the liver, thereby reversing ethanol-induced liver damage. Furthermore, chronic ethanol consumption increased TG concentrations, leading to their accumulation as lipid droplets within hepatocytes and hepatic steatosis [50]. These effects of ethanol-induced fat accumulation were confirmed by measuring the TG concentration in the liver tissue following MC-100093 treatment. Notably, MC-100093 significantly decreased hepatic TG levels in both male and female P rats. In line with these findings, MC-100093 has previously been shown to reduce lipid droplets in the liver of male P rats, suggesting its efficacy in reversing ethanol-induced liver steatosis [27]. However, because MC-100093 also reduced voluntary ethanol intake, the improvement in hepatic triglyceride accumulation cannot be fully attributed to a direct hepatoprotective action of the compound and may partly reflect reduced cumulative ethanol exposure (see Limitations).

Chronic ethanol consumption can activate inflammatory factors (e.g., IL-6, TNF-α, and IL-1β) and promote the infiltration of inflammatory cells [51,52,53]. Ethanol-induced ROS can activate Kupffer cells (KCs), which in turn drive activation of the immune system via several cytokines, including TNF-α, IL-6, and IL-1β [54,55]. Activation of TNF-α and IL-1β induced liver injury, and treatment with anti-TNF-α [56] and an IL-1 receptor antagonist (IL-1Ra) reversed this injury [20]. Notably, treatment with MC-100093 decreased the levels of these inflammatory cytokines in the livers of P rats of both sexes, thereby attenuating the liver injury. In addition, treatment with MC-100093 decreased p-STAT-3 expression in the liver. STAT-3 has been linked to the regulation of inflammation and fibrosis, which are activated by IL-6 and other inflammatory factors [57]. Alcohol-induced liver inflammation can lead to further damage, resulting in fibrosis [58]. Furthermore, MC-100093 was associated with decreased COL-1 expression, thereby mitigating the fibrosis induced by six weeks of ethanol consumption, an effect also notable in the histological analysis of liver sections stained with MT. Therefore, our findings add to the accumulating evidence that inhibition of STAT-3 [59,60] and reversing the increases in the levels of TNF-α and IL-1β [20,56] are associated with the amelioration of fibrosis and inflammation in the liver. In this study, model establishment was demonstrated by the behavioral response to drug treatment, as MC-100093 significantly reduced ethanol drinking in P rats. The liver findings reported here reflect the hepatic outcomes of this reduced ethanol intake, through reversal of ethanol-induced changes in glutamate transporters, inflammation, and fibrosis. Therefore, ethanol-induced glutamate release can activate hepatic stellate cells and Kupffer cells through mGluR5 signaling, driving cytokine release and fibrogenesis, and this pathway may be linked to reduced drinking, normalization of GLT-1 and xCT expression, and the improved liver pathological outcomes observed with MC-100093 treatment [21].

The present study has several limitations. First, this study lacks an ethanol-intake-matched or pair-drinking control group, which prevents distinguishing a direct hepatoprotective effect of MC-100093 from an indirect effect secondary to reduced ethanol consumption. Thus, the observed improvements in hepatic steatosis, inflammation, STAT3 activation, collagen deposition, and associated protein expression may be attributable, in part, to lower cumulative ethanol exposure and can be interpreted as treatment-associated rather than direct hepatoprotective effects. Second, because protein expression was measured in whole-liver homogenates, the observed changes cannot be localized to hepatocytes, Kupffer cells, hepatic stellate cells, or other hepatic cell types. Moreover, transporter expression was not supported by functional measurements, such as hepatic glutamate uptake, cystine/glutamate exchange, membrane localization, or hepatic glutamate and glutathione levels. Finally, liver weight, liver-to-body-weight ratio, and plasma ALT and AST activities were not measured. Future studies incorporating ethanol-intake-matched controls, hepatic drug-exposure measurements, isolated or cell-specific liver models, transporter-functional assays, and histopathological scoring are needed to define the hepatic effects and mechanisms associated with MC-100093 treatment.

## 5. Conclusions

Taken together, these findings indicate that MC-100093 significantly alleviated ethanol-induced increases in hepatic triglyceride levels, inflammation, and fibrosis by reducing ethanol consumption and modulating glutamate transporters, including xCT and GLT-1. Additionally, the results show that MC-100093 was effective in preventing further liver injury by attenuating ethanol intake and reversing ethanol-induced liver damage. It is important to recognize that MC-100093 influenced both the behavioral aspects of ethanol intake and the molecular regulation of the main glutamate transporters, GLT-1 and xCT. Further studies are warranted to explore the role of glutamate transporters in the liver-brain axis related to AUD.

## Data Availability

The original contributions presented in this study are included in the article. Further inquiries can be directed at the corresponding author.

## Abbreviations

The following abbreviations are used in this manuscript:

AFL	Alcohol-related fatty liver
ALD	Alcohol-related liver disease
ASH	alcohol-related steatohepatitis
AUD	Alcohol use disorder
COL-1	Collagen type 1
CYP	Cytochrome P
EAAT2	Excitatory amino acid transporter 2
GLT-1	Glutamate Transporter-1
H&E	Hematoxylin and eosin
HSCs	Hepatic stellate cells
IL-1β	Interleukin-1β
IL-1Ra	Interleukin-Ra
IL-6	Interleukin-6
KCs	Kupffer cells
mGluR5	Metabotropic glutamate receptor 5
MT	Masson’s trichrome
ORO	Oil Red O
P rats	Alcohol preferring rats
ROS	Reactive oxygen species
STAT3	Signal transducer and activator of transcription 3
TBST	Tris-buffered saline Tween-20
TG	Triglyceride
TNF-α	Tumor necrosis factor alpha
xCT	Cystine/glutamate antiporter
